# Antioxidant and anti-inflammatory effects of zinc. Zinc-dependent NF-κB signaling

**DOI:** 10.1007/s10787-017-0309-4

**Published:** 2017-01-12

**Authors:** Magdalena Jarosz, Magdalena Olbert, Gabriela Wyszogrodzka, Katarzyna Młyniec, Tadeusz Librowski

**Affiliations:** 1 Department of Radioligands, Jagiellonian University Medical College, Medyczna 9, 30-688 Krakow, Poland; 2 Department of Pharmaceutical Technology and Biopharmaceutics, Jagiellonian University Medical College, Medyczna 9, 30-688 Krakow, Poland; 3 Department of Pharmacobiology, Jagiellonian University Medical College, Medyczna 9, 30-688 Krakow, Poland

**Keywords:** Zinc, Oxidative stress, Inflammation, NF-κB signaling, Protein A20, ZIP8

## Abstract

Zinc is a nutritionally fundamental trace element, essential to the structure and function of numerous macromolecules, including enzymes regulating cellular processes and cellular signaling pathways. The mineral modulates immune response and exhibits antioxidant and anti-inflammatory activity. Zinc retards oxidative processes on a long-term basis by inducing the expression of metallothioneins. These metal-binding cysteine-rich proteins are responsible for maintaining zinc-related cell homeostasis and act as potent electrophilic scavengers and cytoprotective agents. Furthermore, zinc increases the activation of antioxidant proteins and enzymes, such as glutathione and catalase. On the other hand, zinc exerts its antioxidant effect via two acute mechanisms, one of which is the stabilization of protein sulfhydryls against oxidation. The second mechanism consists in antagonizing transition metal-catalyzed reactions. Zinc can exchange redox active metals, such as copper and iron, in certain binding sites and attenuate cellular site-specific oxidative injury. Studies have demonstrated that physiological reconstitution of zinc restrains immune activation, whereas zinc deficiency, in the setting of severe infection, provokes a systemic increase in NF-κB activation. In vitro studies have shown that zinc decreases NF-κB activation and its target genes, such as TNF-α and IL-1β, and increases the gene expression of A20 and PPAR-α, the two zinc finger proteins with anti-inflammatory properties. Alternative NF-κB inhibitory mechanism is initiated by the inhibition of cyclic nucleotide phosphodiesterase, whereas another presumed mechanism consists in inhibition of IκB kinase in response to infection by zinc ions that have been imported into cells by ZIP8.

## Zinc biology

In 1963, nearly a century after demonstrating the essentiality of zinc (Zn) for the growth of *Aspergillus niger* (Raulin 1869), zinc deficiency in man was recognized and described by Prasad et al. (1963). Since then, the impact of zinc on human health has been thoroughly investigated. To date, numerous studies have shown that zinc, rather than being a toxic transition metal, is a nutritionally fundamental non-toxic trace mineral (Fosmire 1990). It is neither cytotoxic, nor carcinogenic, mutagenic or teratogenic (Léonard et al. 1986). In addition, the reported zinc intoxications are rare and related primarily to copper deficiency (Plum et al. 2010; Młyniec et al. 2015a; Merza et al. 2015). On the other hand, deregulated homeostasis and even marginal zinc deficiency pose significant risk to healthy individuals.

Zinc, after iron, is second most prevalent trace element in the human body (Vašák and Hasler 2000). The total amount of zinc in adults is about 1.4–2.3 g, but its content varies significantly between tissues. 85% of zinc is localized in the muscles and bones, 11% in the skin and liver, and the remaining 4% in other tissues of the body (Calesnick and Dinan 1988). Highest concentrations of zinc have been determined in the retina and choroid of the eye, followed by the prostate, bones, liver, and kidneys (Tipton et al. 1965; Karcioglu 1982). Since zinc is present in each organ, tissue, and fluid of the body, its deficiency proves crucial for human well-being. Marginal-to-moderate deficiency leads to growth retardation, poor appetite, impaired immunity, enhanced oxidative stress, and increased generation of inflammatory cytokines. Further symptoms include skin reactions, delayed wound healing, and declined reproductive capacity (Prasad et al. 1963, 2001, 2014b; Tapiero and Tew 2003; Lansdown et al. 2007). Adequate intake is of great importance also to neuropsychological performance. Zinc deficiency is increasingly associated with mental lethargy, cognitive impairment, symptoms of depression, and Alzheimerʼs disease (Adlard and Bush 2011; Szewczyk et al. 2011a, b; Gower-Winter and Levenson 2012; Maes et al. 2012; Młyniec et al. 2014, 2015b, 2015). Most severe clinical manifestations of zinc deficiency are observed in acrodermatitis enteropathica (AE). This rare inheritable autosomal recessive metabolic disorder may become fatal if not recognized and treated instantly with zinc (Vallee and Falchuk 1993). To fully appreciate the significance of zinc to human health, one needs to be aware of the great number of biological processes requiring zinc-containing proteins.

The element is essential to the structure and function of about 2800 macromolecules and over 300 enzymes. It is a component of about 10% of human proteins, including transcription factors and key enzymes regulating cellular processes and cellular signaling pathways (Rink and Gabriel 2001; Andreini et al. 2006). Most of the zinc-containing enzymes catalyze hydrolysis reactions, but representatives of all enzyme classes are known (Vallee and Falchuk 1993). The ion is critically responsible for cell proliferation, differentiation, and apoptosis. The intermediary metabolism, DNA synthesis, reproduction, vision, taste, and cognition are all zinc-dependent. Studies have shown that zinc safeguards DNA integrity and its deficiency can impair the function of zinc-dependent proteins involved in the DNA damage response (Yan et al. 2008). Moreover, a growing body of evidence suggests that zinc deficiency increases the concentrations of inflammatory cytokines and oxidative stress, induces apoptosis, and causes cell dysfunction. The element plays, therefore, a preventive role against free radical formation and protects biological structures from injury during inflammatory processes (Powell 2000; Tapiero and Tew 2003; Stefanidou et al. 2006; Chasapis et al. 2012).

Enumerating impressive structural, catalytic, and regulatory functions of zinc is beyond the scope of this article. Nevertheless, the antioxidant and anti-inflammatory properties of zinc are discussed more particularly later.

## Zinc homeostasis

The current RDAs (Recommended Dietary Allowances) for zinc given by Institute of Medicine are 11 mg/day for males and 8 mg/day for females (Institute of Medicine (US) Panel on Micronutrients 2001). However, individual requirements may vary widely depending on numerous factors influencing zinc uptake and excretion, such as age, stress, and illness conditions or applied diet (European Commission, Health and Consumer protection directorate general 2003). Zinc is the element with a minor plasma pool (13.8–22.9 µmol/L) and a rapid turnover (Bonaventura et al. 2015). There is no store for zinc in the body and the gastrointestinal tract is the main site for regulation of its balance (Tapiero and Tew 2003). In healthy subjects, zinc homeostasis can be efficiently maintained under conditions of zinc excess or deprivation over a wide range of dietary intake through modulation of its intestinal uptake and excretion (Jackson et al. 1984; Hambidge et al. 2010). Zinc is absorbed primarily in the duodenum, ileum, and jejunum by a carrier-mediated process or more rarely by passive diffusion (Vallee and Falchuk 1993; Sian et al. 1993; Tapiero and Tew 2003). After entering the duodenum within 3 h zinc passes into the bloodstream. Distribution occurs via the serum, where about 84% of zinc is bound to albumin, 15% to α2-globulins, and 1% to amino acids (Chesters and Will 1981; Foote and Delves 1984). In multicellular organisms, virtually, all zinc is intracellular. 30–40% of zinc is localized in the nucleus, 50% in the cytosol, organelles, and specialized vesicles, and the remainder is associated with cell membranes (Vallee and Falchuk 1993). The cellular homeostasis of zinc and its intracellular distribution is controlled by specialized transport and binding proteins. Zn^2+^ transport through lipid bilayers is mediated by two protein families; 14 ZIP (zinc importer family, SLC 39A) and 10 ZNT (zinc transporter family, SLC 30A) transporters (Lichten and Cousins 2009). ZNT proteins generally transport zinc ions out of the cytosol, whereas ZIP proteins import them from cellular compartments or the extracellular space into the cytosol. The two families of transporters precisely control zinc availability due to tissue specific expression profiles and different subcellular localizations.

Human homeostatic mechanisms maintain plasma zinc within the reference range of approximately 10–18 µmol/L (Foster and Samman 2012). However, an interpretation of serum zinc levels may not be apparent. Plasma zinc represents only 0,1% of total body zinc and is an insensitive marker for zinc deficiency. Immune cells may be the first to respond to zinc deficiency even before plasma zinc. Moreover, its biological variation is high and only a change above 30% is likely to be significant. Finally, hypozincemia can be caused by factors unrelated to zinc status, such as ongoing acute phase response (APR) or hypoalbuminemia (Livingstone 2015). Inflammatory processes are associated with remarkable changes in zinc homeostasis. The APR rapidly decreases the serum zinc concentration due to the redistribution of zinc from plasma into organs, predominantly the liver. The proinflammatory cytokine IL-6 has been shown to up-regulate ZIP14 in mouse liver (Liuzzi et al. 2005). Such decline in plasma zinc has been suggested to be an adaptive response intended to deprive invading pathogens of zinc. At the same time, macrophages increase the concentrations of zinc to intoxicate phagocytosed microorganisms (Shankar and Prasad 1998; Haase and Rink 2014). Moreover, hypozincemia may be the consequence of chelation of zinc by the zinc and calcium binding S-100 protein calprotectin, which is released by leukocytes. Calprotectin has been shown to suppress the reproduction of bacteria and *Candida albicans* (Sohnle et al. 2000). On the other hand, increased intracellular zinc serves a role in energy metabolism, provides efficient neutralization of reactive nitrogen and oxygen species, and guarantees proper synthesis of proteins and more specifically the synthesis of acute phase proteins in the liver (Powanda et al. 1973; Haase and Rink 2009). Therefore, zinc redistribution during inflammation may serve multiple purposes.

Finally, zinc homeostasis maintenance is supported by intracellular zinc binding proteins. Up to 20% of intracellular zinc is complexed by metallothioneins (MTs). These ubiquitous cysteine-rich proteins with a low-molecular weight bind up to seven zinc ions, acting as a cellular zinc buffer. They play a significant role in metal uptake, distribution, storage, and release (Cousins 1985; Vašák and Hasler 2000). Maintaining physiological concentrations of zinc and its tight control by MTs in each cell of the body is necessary to avoid oxidative stress, since not only zinc deficiency but also zinc overload are pro-oxidant conditions (due to inhibition of mitochondrial respiration and antioxidant enzymes) (Skulachev et al. 1967; Maret 2000). In principle, the increase in the amount of zinc in applied diet results in increase in MT concentration in enterocytes. In addition, in turn, the higher MT levels, the less zinc is further absorbed from gastrointestinal tract (Sullivan et al. 1998). By binding zinc and regulating zinc absorption, MT protects the cell from its overload and releases the element when necessary.

## Zinc and metallothioneins

Metallothioneins are metal-binding proteins with high affinity to divalent trace minerals, such as zinc and copper, as well as to toxic cadmium and mercury ions. Their presumed functions in the physiological condition include heavy metal detoxification, metal storage, and donation to target apometalloproteins (particularly to zinc finger proteins and newly synthesized apoenzymes) (Cousins 1985; Coyle et al. 2002; Kondoh et al. 2003). Serving as both zinc acceptor and zinc donor and thereby controlling the concentration of readily available zinc ions appears to be the major and most important role of MT.

The cluster structure of the protein with two domains, in each of which zinc ions are bound tetrahedrally to cysteines, precludes access of ligands to zinc. Zinc/sulphur cluster with low redox potential is very sensitive to changes of cellular redox state, and therefore, sulfhydryl groups of MTs are readily oxidized by a number of mild cellular oxidants with concomitant release of zinc. In brief, a shift to more oxidizing conditions releases zinc, whereas a shift to more reducing environment leads to its binding (Maret 1995; Maret and Vallee 1998). Zinc ions, only rapidly released by MTs, are able to play its relevant function against oxidative stress and participate in immune responses. MTs are ipso facto the link between zinc and cellular redox status of the cell (Krezel and Maret 2007). Furthermore, as repeatedly confirmed in the previous studies, MTs themselves act as potent electrophilic scavengers and cytoprotective agents against oxidative and inflammatory injury (Andrews 2000; Kang et al. 2015). They are able to capture a wide range of reactive oxygen species (ROS), including superoxide, hydrogen peroxide, hydroxyl radicals, and nitric oxide (Sato and Kondoh 2002; Ruttkay-Nedecky et al. 2013). It has been shown that the ability of MTs to scavenge hydroxyl radicals is 300× higher than that of glutathione, the most abundant antioxidant in the cytosol (Sato 1992). Thus, under physiological conditions, MTs can efficiently protect biological structures and DNA from the oxidative damage. Concerns may be raised about the roles of MTs under pathophysiological conditions.

Since proinflammatory cytokines, such as tumor necrosis factor TNF, IL-1, IL-6, and interferon-γ, do induce hepatic MT gene expression in vivo, the role of MT in inflammatory processes needed to be examined (Waelput et al. 2001; Inoue et al. 2009). Various types of inflammatory conditions have been studied (including allergic, oxidative and LPS-related), in which MT has been shown to protect against ovalbumin-induced allergic airway inflammation, against ozone-induced lung inflammation, and against coagulatory and fibrinolytic disturbances and multiple organ damage induced by lipopolysaccharide (LPS). Antioxidant effects of MT have also been confirmed in response to exposure to radiation, ethanol, and toxic anticancer drugs (Powell 2000). However, conflicting results were also reported. Kimura et al. showed that d-galactosamine (GalN)-sensitized MT-null mice are more sensitive to LPS-induced lethality presumably through the reduction of protective α1-acid glycoprotein (AGP) than wild-type mice, whereas Waelput et al. observed significantly higher survival in MT-null mice compared to wild-type mice in TNF-induced lethal shock (Kimura et al. 2001; Waelput et al. 2001). Moreover, it was found that TNF-α is likely to act as a final mediator of endotoxin action in a sequence of events characterized by but not limited to reactive oxygen species formation (Tiegs et al. 1989), which may partly explain the protection against LPS/GalN but not against TNF/GalN by antioxidants. The question then arises why MT-null animals were more resistant to TNF lethality in comparison with wild-type and MT-overexpressing ones. The possible interpretation of these findings is that increased MT expression contributes to rapid redistribution of tissue zinc levels, which may represent an acute disruption of zinc homeostasis (Wong et al. 2007). Interestingly, Waelput et al. showed that zinc depletion increased the sensitivity of both MT-null and wild-type mice to TNF toxicity and that zinc sulphate-pretreated animals were significantly protected against TNF. The authors ascribe the zinc mediated protection against TNF to metal responsive genes and more specifically to hsp70 gene, which is strongly induced in jejunum after zinc sulphate treatment (Waelput et al. 2001). Although the findings have significant implications for the understanding of the substantial role of MT in stress conditions, inflammation and infection, further studies will be necessary to reveal the different roles of MT under pathophysiological conditions.

## Zinc in oxidative stress and inflammation

Oxidative stress underlies the molecular mechanisms responsible for the development of many inflammatory diseases, such as atherosclerosis, diabetes mellitus, rheumatoid arthritis, cancer, and neurodegeneration (Valko et al. 2007). It occurs when cellular antioxidant systems prove insufficient to remove increased ROS levels. Although ROS play beneficial role in the immune response to infection, their excess causes lipid peroxidation and damage to proteins and nucleic acids (Castro and Freeman 2001).

Not only oxidative stress may lead to the inflammatory response, but inflammation itself may provoke free radical formation. A large amount of ROS and RNS is generated by phagocytic cells, neutrophils, and macrophages, as part of their essential role in host defense, in a mechanism dependent from oxygen, also called the oxidative outburst. The major intracellular sites of ROS production in eukaryotic cells are mitochondrial electron transport chain, peroxisomal long-chain fatty acid oxidation, and respiratory burst mainly via activation of NADPH oxidases. In addition, other enzymes, including cytochrome P450 monooxygenase, nitric oxide synthase (NOS), xanthine oxidase, cyclooxygenase (COX), and lipoxygenase (LOX), generate ROS through their enzymatic reaction cycles (Bhattacharyya et al. 2014; Holmström and Finkel 2014). Furthermore, free radical chain reactions may be induced by transition metals and in response to many exogenous factors, such as pollutants, ultraviolet radiation, cigarette smoking, alcohol, and drugs, such as nonsteroidal anti-inflammatory drugs (NSAIDs). Chronic infections and inflammatory disorders also provoke the increased production of free radicals (Bhattacharyya et al. 2014; Sharma et al. 2014). Therefore, to combat ROS, cells are equipped with potent enzymatic and non-enzymatic antioxidant defences.

Non-enzymatic antioxidants include glutathione (GSH), thioredoxin (Trx), and melatonin. Antioxidant enzymatic mechanisms involve enzymes, such as superoxide dismutase (SOD), glutathione peroxidase (GPX), glutathione reductase (GR), catalase (CAT), and heme oxygenase (HO) (Castro and Freeman 2001; Rahman 2007; Bhattacharyya et al. 2014). From all above mentioned, SOD and catalase provide major antioxidant defences against ROS. Superoxide dismutase exists in several isoforms. Zinc is a co-factor of cytosolic and extracellular Zn/Cu SOD enzyme, which acts as an ROS scavenger by catalyzing the dismutation of O_2_^−^ radical into the less harmful O_2_ and H_2_O_2_ (Mariani et al. 2008). Except against oxidative stress, the efficacy of Zn/Cu SOD is also crucial for the resolution of inflammation. Neutrophils recruited to the inflammation sites generate ROS, protease enzymes, and chemokines. Consequently, the healthy tissue is being damaged and further influx of inflammatory cells is maintained. For the reduction of inflammation, activated neutrophils must be removed safely by apoptosis. As H_2_O_2_ has been suggested to be a possible major mediator of ROS-induced neutrophil apoptosis in a caspase-dependent manner, the proper functioning of SOD enzyme contributes to the regulation of neutrophil apoptosis and neutrophil-mediated tissue injury (Yasui et al. 2005, 2006). The more H_2_O_2_ produced by Zn/Cu SOD, and the more neutrophils undergo apoptosis. Thus, zinc, as a component of SOD, procaspase-3, and other enzymes involved in neutrophil apoptosis, plays an important role during inflammatory response (Zalewski et al. 1993; Ho et al. 2004). Moreover, in a study by Goel et al. (2005), zinc treatment to chlorpyriphos-intoxicated animals normalized the otherwise increased levels of lipid peroxidation to within normal levels. Zinc treatment to these animals elevated the levels of GSH, catalase, and detoxifying glutathione-S-transferase (GST). Zinc has also been proven to exhibit its antioxidant effect by inducing heme oxygenase and inhibiting NADPH oxidase (Tapiero and Tew 2003; Prasad 2014b).

The critical transcription factor that regulates the expression of genes encoding above mentioned antioxidant and detoxifying molecules (GSH, SOD, GST, HO-1), nuclear factor erythroid 2-related factor 2 (Nrf2), has been proven to be up-regulated by zinc. Studies revealed significantly increased oxidative damage and decreased Nrf2 expression in zinc-deficient mice (Zhao et al. 2010), as well as increased HO-1 mRNA and Nfr2 protein levels in human colon cancer HCT 116 cells in response to high concentrations of zinc (Smith and Loo 2012). It has also been shown that zinc can protect endothelial cells from hydrogen peroxide via Nrf2-dependent stimulation of glutathione biosynthesis (Cortese et al. 2008). Since zinc up-regulates Nrf2, also through this pathway, it contributes to the regulation of oxidative stress-induced cellular damage.

The antioxidant mechanisms, which involve zinc, can be divided into acute and chronic. Chronic effects in response to long-term exposure to zinc consist in induction of some other ultimate antioxidant substances, above all, previously described metallothioneins (MTs) (Cousins 1985; Powell 2000). Chronic zinc deficiency impairs the activity of MTs and renders the organism more susceptible to injury induced by various oxidative stressors. On the other hand, zinc retards oxidative processes via two acute mechanisms, one of which is the stabilization of protein sulfhydryls against oxidation (Bray and Bettger 1990; Powell 2000). There are three ways proposed by Gibbs et al. (1985), in which zinc reduces sulfhydryl reactivity. First, zinc binds directly to the thiol group. Second, it creates steric hindrance, by binding in the close proximity to the sulfhydryl group of the protein. Third, it changes the conformation of the protein, by binding to the other site of the protein. The most extensively studied enzyme for sulfhydryl protection by zinc is δ-aminolevulinate dehydratase, which catalyzes the formation of the pyrrole porphobilinogen. The presence of the metal prevents enzyme thiol oxidation and disulphide formation. Contrary, the removal of zinc increases sulfhydryl reactivity resulting in the loss of dehydratase activity (Powell 2000; Tapiero and Tew 2003). Other examples of sulfhydryl-containing proteins protected by zinc are DNA zinc-binding proteins (zinc fingers), alanyl tRNA synthetase, tubulin, and dihydroorotase (Mocchegiani et al. 2000; Rink and Gabriel 2001; Pace and Weerapana 2014).

The second acute antioxidant effect of zinc consists in antagonizing transition metal-catalyzed reactions, such as reduction of ·OH formation from H_2_O_2_ and O_2_^−^ (Powell 2000). Redox-active transition metals have been demonstrated to catalyze formation of radicals, mainly through Fenton reaction (Jomova and Valko 2011). Any ·OH formed in this reaction attacks adjacent structures and causes severe localized damage. The damage is all the greater because in physiological media copper and iron tend to associate with specific cellular components, such as nucleotides and glucose for iron or DNA, carbohydrates, enzymes, and proteins for copper. Transition metals bound to molecules form the coordination complex, which subsequently, reacts with H_2_O_2_ and forms ·OH radical. The radical can then react with hydrogen attached to the carboxyl group of the molecule, thereby changing its properties. These sites serve as loci for repetitive radical formation through repeated redox cycling of the metals. Transition metal-induced free radical chain reactions lead to lipid peroxidation, DNA, and protein damage. Both iron and copper play a critical role in initiation and propagation of lipid peroxidation, which destructs lipid bilayers. Overall, redox-active transition metals associated with cellular components establish a site for the repetitive formation of ·OH radicals. Only high affinity chelators or some chemically similar, yet redox-inactive agents can antagonize the formation of ·OH or shift the formation site to less critical one. By virtue of similarities, zinc can exchange copper and iron in certain binding sites and attenuate cellular site-specific oxidative injury. The metal is, therefore, capable of reducing postischemic injury to a variety of tissues and organs, such as stomach, kidney, intestine, retina, and brain (Powell 2000; Tapiero and Tew 2003).

## Zinc and immunity

The profound effect of zinc on innate and adaptive immunity is undisputable. Zinc is critical for maintaining membrane barrier structure and function. Its deficiency causes damage to epidermal cells and to the linings of the gastrointestinal and pulmonary tracts, what may facilitate the entrance of potential pathogens and noxious agents into the body (Shankar and Prasad 1998). The first cells, which recognize and eliminate invading pathogens, are cells of the innate immune system, notably polymorphonuclear cells (PMNs), macrophages, and natural killer (NK) cells. Zinc deficiency leads to reduced PMN chemotaxis and decreased phagocytosis, while zinc supplementation has the opposite effect. The destruction of pathogens after phagocytosis relies, among others, upon the activity of NADPH oxidase, which may be inhibited by both zinc deficiency and zinc excess. Moreover, zinc augments monocyte adhesion to endothelial cells in vitro and affects production of proinflammatory cytokines, such as interleukins IL-1β, IL-6, and TNF-α. The element is also involved in recognition of major histocompatibility complex (MHC) class I by NK cells, and the lytic activity of NK cells is affected during zinc depletion. In vitro, moderate zinc supplementation increases the differentiation of CD34 + cells toward NK cells and their cytotoxic activity. Furthermore, in terms of adaptive immunity, zinc deficiency is responsible for thymic atrophy and subsequent T-cell lymphopenia as well as reduction of B cells, affecting antibody production. Zinc is also crucial for the balance between the different T-cell subsets (Foster and Samman 2012; Haase and Rink 2014; Bonaventura et al. 2015). This theme is thoroughly presented by Shankar and Prasad (1998).

Simultaneously, antimicrobial secretory molecules also contribute to innate immunity of the host. Zinc supplementation was shown to improve mucosal innate immunity through stimulation of antimicrobial peptide secretion from intestinal epithelium cells. Notably, the production of the antimicrobial peptide LL-37 from Caco-2 cells (human epithelial colorectal adenocarcinoma cell line) was enhanced by zinc in a dose- and time-dependent manner, showing beneficial effects against infectious diseases, particularly diarrhoea (Talukder et al. 2011). The cathelicidin LL-37 was shown to exert a potent antimicrobial activity against a variety of bacteria, including *Pseudomonas aeruginosa*, *staphylococcal* species and *Escherichia coli* as well as against viruses (HSV-1) and fungi, such as *Candida albicans* (Gordon et al. 2005). Another beneficial effect of zinc on secretory molecules concerns its role in bactericidal activity of human peptidoglycan recognition proteins (PGLYRPs). These are secreted innate immunity pattern recognition molecules with zinc-dependent effector function, acting mainly against Gram-positive and negative bacteria (Wang et al. 2007). Recently, the outer membrane receptor in *Neisseria meningitidis* was shown to be involved in zinc acquisition of bacteria. The receptor is produced under zinc limitation and is believed to control zinc uptake. Homologues of this receptor protein are present in many other Gram-negative pathogens, particularly in those residing in the respiratory tract (Stork et al. 2010). What these findings clearly illustrate is that zinc plays its role in basically all aspects of immunity.

A number of animal studies have been conducted to evaluate the effect of zinc on survival in the setting of lethal infections. In general, the experiments involved zinc sufficient adult subjects that received lethal quantities of different infectious agents. Either prior to, simultaneously with or after an endotoxin injection animals were injected with zinc. Various zinc salts and unequal doses were administered to the animals, what makes a direct comparison of study findings more difficult. In addition, different routes of administration of both endotoxin and zinc were applied, i.e., intraperitoneal or intravenous. Nevertheless, zinc significantly improved animal survival when administered before or coincident with the challenge. Intraperitoneal route of administration of zinc salt provided protection from mortality and necrotic lesions in the liver after a lethal quantity of intraperitoneally administered *Salmonella typhimurium* endotoxin (Sobocinski et al. 1977a). The authors perceive the reason for such protection in the ability of zinc to decrease the absorption of endotoxin from the peritoneal cavity with its subsequent hepatic uptake. Similarly, in a study by Tocco-Bradley and Kluger, prevention of infection-induced hypozincemia enhanced rather than reduced survival rate in animals injected intravenously with *S. typhimurium* (Tocco-Bradley and Kluger 1984). Contradictory results were obtained by Sobocinski and colleagues in rats infected with live *S. typhimurium* (but not with *Francisella tularensis* and *Streptococcus pneumoniae*), i.e., the incidence of mortality in infected rats was enhanced after treatment with zinc chloride 1 h prior to bacterial challenge (Sobocinski et al. 1977b). It should be noted, however, that plasma zinc levels during the infection were raised high above physiological levels and that zinc toxicity may have played a role in increased mortality. Apparently, the protective effect of zinc during an infection depends on the infectious agent itself, zinc levels in the host prior to infection, the concentration of zinc administered, route of administration, and time of onset of administration.

Worth mentioning are also studies that evaluated the resistance of zinc deficient animals to infectious diseases. It has been repeatedly proven that zinc deficiency results in suppressed immune responses and increased susceptibility to infectious agents, including *F. tularensis* (Pekarek et al. 1977), *Listeria monocytogenes* (Coghlan et al. 1988), Salmonella enteritidis (Kidd et al. 1994), *Mycobacterium tuberculosis* (McMurray et al. 1990), and many viruses, protozoan parasites, and eukaryotes (Shankar and Prasad 1998). The results of these studies acknowledged that zinc deficiency in animals are responsible for their poorer performance during endotoxin challenge due to the delay in production of protective antibodies. All above examples clearly show that zinc affects the immune system in a multi-faceted way.

Several studies have demonstrated the beneficial effects of zinc supplementation on infectious diseases in humans. In double-blind, placebo-controlled trials daily zinc supplementation has been shown to prevent and treat diarrhoea. Zinc lozenges were shown to decrease the duration of common cold. Risk for respiratory infections was correlated with zinc deficiency. Although there is evidence suggesting a link between infection and zinc deficiency across several other infectious diseases, including pneumonia, malaria, HIV, and tuberculosis, more research is needed to evaluate the actual effect of zinc supplementation on the progression of these diseases. In populations where dietary zinc is inadequate, zinc deficiency increases susceptibility for infection and its duration.

## Zinc and NF-κB pathway

There are many pathways involved in the inflammatory processes that occur in cells. Modulation of these routes is necessary to provide the adequate response of the organism to various stimuli, such as stress, cytokines, free radicals, oxidized LDL, or bacterial/viral antigens. The nuclear factor kappa-light-chain-enhancer of activated B cells (NF-κB) signaling pathway is one of the main inflammatory pathways, which regulate the genes controlling apoptosis, cell adhesion, proliferation, tissue remodeling, the innate and adaptive immune responses, inflammatory processes, and cellular-stress responses. NF-κB, therefore, influences the expression of proinflammatory cytokines (e.g., IL-1β, IL-6, IL-8, TNF-α, and MCP-1), chemokines, acute phase proteins (CRP and fibrinogen), matrix metalloproteinases (MMPs), adhesion molecules, growth factors, and other factors involved in inflammatory response, such as COX-2 and iNOS (Lawrence 2009; Ghosh and Hayden 2012; Prasad 2014a). The NF-κB proteins rank among the most versatile regulators of gene expression.

The mammalian NF-κB protein family is composed of five members: p50/p105, p52/p100, RelA (p65), c-Rel, and RelB, and different NF-κB complexes are formed from their homo- and heterodimers. NF-κB proteins are not synthesized de novo, but are present in the cytoplasm in non-active form. Their transcriptional activity is silenced by a family of inhibitory proteins known as inhibitors of NF-κB (IκBs); i.e., IκBα, IκBβ, IκBγ, IκBε, Bcl-3, and the precursor proteins p100 and p105. The NF-κB protein family is characterized by the presence of a conserved N-terminal 300 amino acid Rel homology domain (RHD) that oversees dimerization, interaction with IκBs, and binding to DNA. The typical NF-κB complex consists of p65–p50 heterodimer and IκBα. NF-κB dimer becomes active when IκB undergoes phosphorylation by the IκB kinase (IKK) complex, which leads to ubiquitination and proteasomal degradation of IκB. As a consequence, released NF-κB translocates freely from the cytoplasm to the nucleus and induces target gene expression (Perkins 2007).

**Fig. 1:**
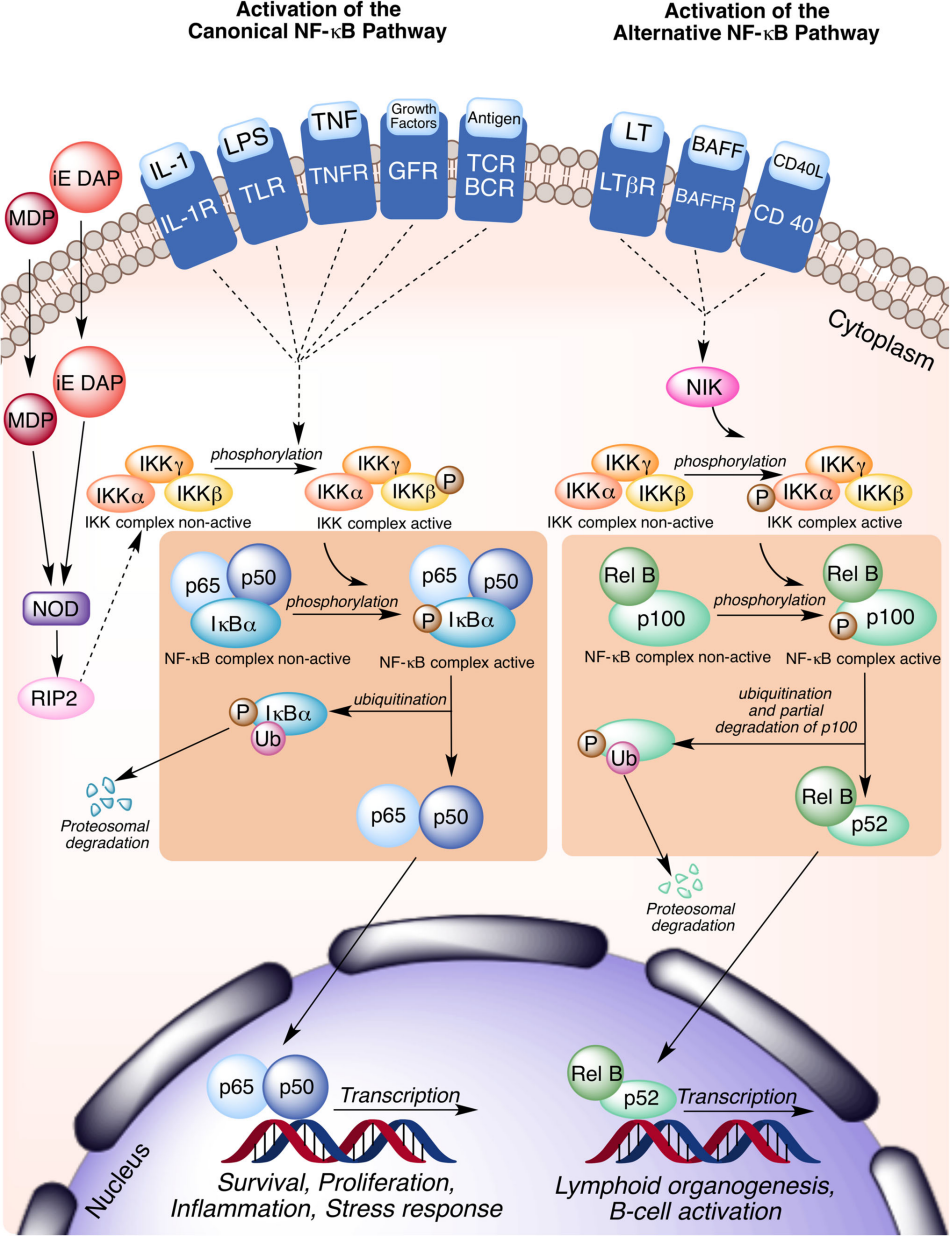
Canonical and alternative pathways for NF-κB activation. The canonical pathway is dependent on activation of IKKβ and is triggered mainly by proinflammatory cytokines, such as tumor necrosis factor-α (TNFα) and interleukin-1 (IL-1), bacterial lipopolysaccharides (LPS), growth factors, and antigens. Activation of this pathway regulates expression of proinflammatory and cell survival genes. The alternative NF-κB pathway is activated by lymphotoxin β (LTβ), CD40 ligand, and B-cell activating factor (BAFF) and results in the activation of IKKα by the NF-κB-inducing kinase (NIK), followed by phosphorylation of the p100 NF-κB subunit by IKKα. Activation of the alternative pathway regulates genes required for lymphoid organogenesis and B-cell activation

Signaling pathways leading to the activation of NF-κB can be divided into classical (canonical) and alternative (non-canonical) (Fig. 1). The common regulatory step in both routes is activation of previously mentioned IKK complex, which is composed of catalytic kinase subunits IKKα and/or IKKβ and the regulatory non-enzymatic scaffold IKKγ (NEMO) protein. The non-canonical NF-κB pathway is triggered by signaling through a subset of receptors, including lymphotoxin-β receptor (LTβR), CD40 receptor, and B-cell activating factor receptor (BAFF-R). It predominantly targets activation of the p52/RelB NF-κB complex by the inducible phosphorylation of p100 by IKKα. Activation of the alternative pathway regulates genes required for lymphoid organogenesis and B-cell activation. Contrary, in the canonical pathway, which relies upon NEMO-IKKβ mediated degradation of IκB, the main IKK activating factors are proinflammatory cytokines, bacterial lipopolysaccharides (LPS), growth factors, and antigens. Inputs for the canonical signaling cascade include the tumor necrosis factor receptor (TNFR), Toll-like receptor family (TLR/IL-1R), T-cell receptors (TCRs), and B-cell receptors (BCRs) (MacEwan 2002; Hayden and Ghosh 2004; Sun 2011; Wang et al. 2012; Ghosh and Hayden 2012; Catrysse et al. 2014). The accurate regulation of NF-κB signaling pathways is an absolute requirement for all cells.

Zinc has been proven to modulate NF-κB pathway. In vitro studies differing in cell types and zinc concentrations used have yielded contradictory observations regarding the effects of zinc on NF-κB activation, indicating that the effects may be cell specific (Foster and Samman 2012). Although some of the studies revealed that zinc ions contribute to signal transduction and are thus at least partly involved in the NF-κB activation (Haase et al. 2008, 2014), a large and growing body of the literature confirms the main role of zinc as a negative regulator of NF-κB pathway. Three possible inhibitory mechanisms have been suggested. One of the mechanism is initiated by the inhibition of cyclic nucleotide phosphodiesterase (PDE), and subsequent elevation of cGMP, cross activation of protein kinase A (PKA), and inhibitory phosphorylation of protein kinase Raf-1. By this mechanism, zinc suppressed LPS-induced activation of IKKβ and NF-κB, and subsequent TNF-α production in human monocytes (von Bülow et al. 2007). Another mechanism exerted by the free ion is related to the direct inhibition of IKK upstream of NF-κB. It was recently suggested that this is the mechanism for NF-κB inhibition by Zn^2+^ that has been imported by ZIP8 into monocytes, macrophages, and lung epithelia during an infection (Liu et al. 2013). Zinc transporter ZIP8 (SLC39A8) is a transcriptional target of NF-κB, described as the most significantly up-regulated transporter in response to cytokines, bacteria, and sepsis. ZIP8 increases cytosolic zinc content by promoting extracellular uptake or release from subcellular organelles. Imported into the cell by ZIP8, thiol-reactive zinc induces NF-κB inhibition downstream from MAPKs by blocking IKK complex. ZIP8 is, therefore, a negative feedback regulator of NF-κB acting through zinc-mediated inhibition of IKK in response to infection (Liu et al. 2013; Gálvez-Peralta et al. 2014). Thirdly and most importantly, zinc affects the expression of protein A20. In TNFR- and TLR-initiated pathways, the zinc-finger protein A20 is the main negative regulator of NF-κB activation.

**Fig. 2:**
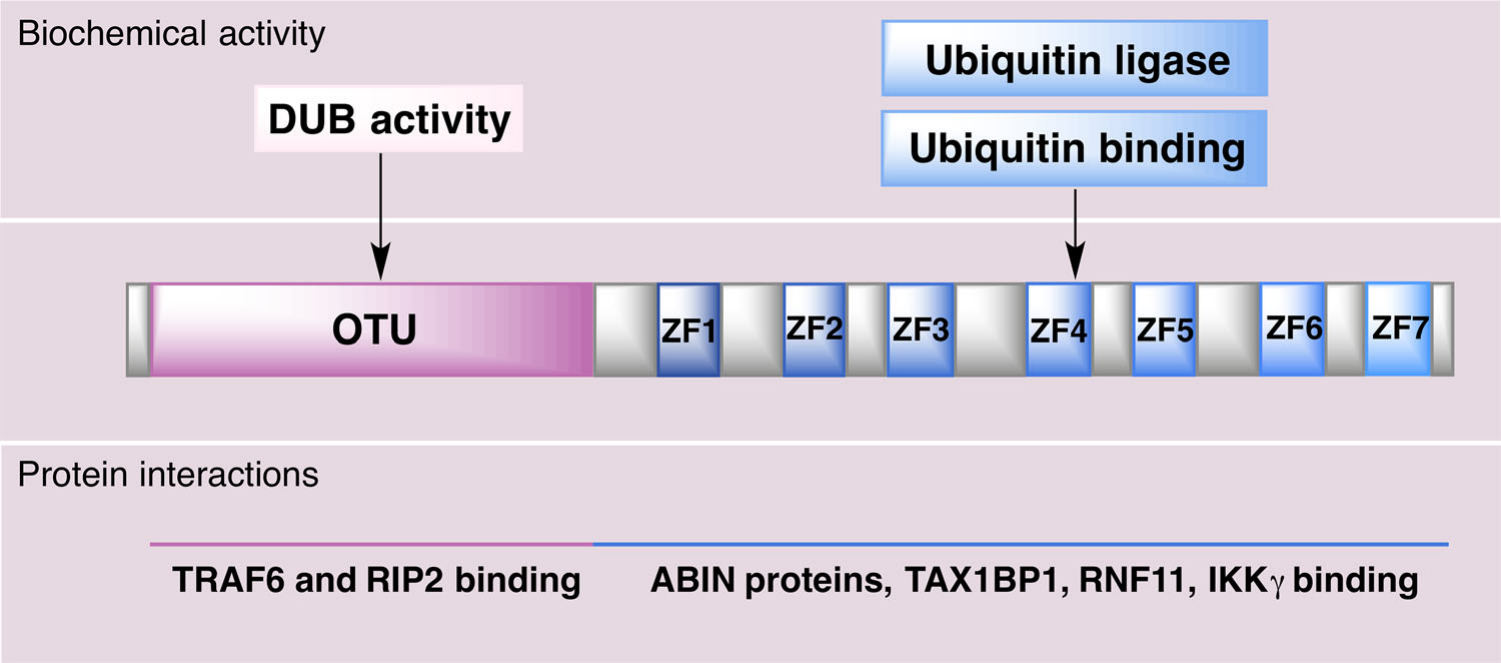
Domain structure of A20. A20 consists of an N-terminal ovarian tumor (OTU) domain and 7C-terminal domain built up by seven zinc fingers (ZF1–ZF7), mediating, respectively, the deubiquitylating (DUB) activity of A20 and its ubiquitin ligase and ubiquitin-binding activity. A20 interacts with substrates, such as receptor-interacting protein 2 (RIP2), and enzymes, such as TNFR-associated factor 6 (TRAF6) via the OTU domain, and with ubiquitin-binding proteins, such as TAX1-binding protein 1 (TAX1BP1), RING-finger protein 11 (RNF11), IκB kinase-γ (IKKγ), A20-binding inhibitor of NF-κB activation 1 (ABIN1), and ABIN2 via the ZF domain

**Fig. 3:**
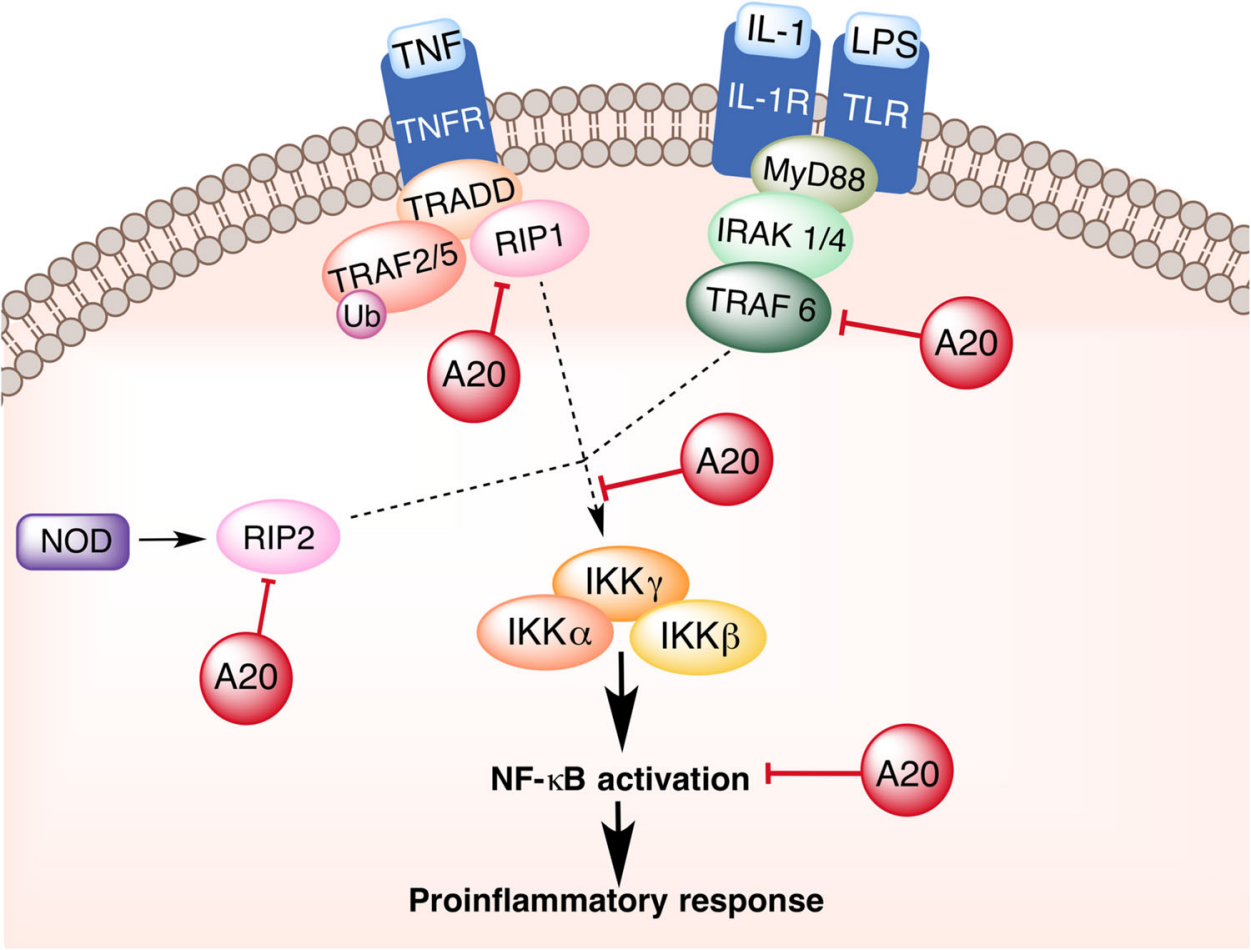
Nuclear factor (NF)-κB regulatory activities of A20. A20 deubiquitinates receptor interacting protein 1 (RIP1), preventing its interaction with NF-κB essential modulator (IKK-γ and NEMO) during TNFR signaling. Moreover, A20 inhibits NF-κB signaling by removing polyubiquitin chains form TNF receptor associated factor 6 (TRAF6) and receptor interacting protein 2 (RIP2) during TLR/IL-1R and NOD signaling, respectively. A20 may interact also with other proteins that bind to ubiquitin

A20 (also known as the TNFα-induced protein 3; TNFAIP3) is a pleiotropically expressed cytoplasmic signaling protein, widely recognized as an anti-inflammatory, NF-κB inhibitory, and antiapoptotic molecule. A20 comprehensively regulates ubiquitin-dependent signals, and in consequence, restricts the duration and intensity of signaling by several proteins involved in NF-κB pathway. Biological activities of A20 vary between individual cells. Whereas its expression is constitutive in thymocytes, mature T cells, and some tumor cells, it is inducible in most tissues. In all cell types, A20 transcription is rapidly induced by multiple NF-κB activating stimuli, including TNFα (Verstrepen et al. 2010; Catrysse et al. 2014). The protein is composed of two domains, an ovarian tumor (OTU) domain with deubiquitinase activity (DUB) and a domain built up by seven zinc fingers, which mediates its ubiquitin ligase and ubiquitin-binding activity (Fig. 2). The ability of A20 to interact with ubiquitin enzyme complexes is critical for modulation of ubiquitin-dependent innate immune signaling cascades, such as those downstream of TNFR1, TLRs, IL-1R, CD40, and NOD-like receptors (NLRs) (Boone et al. 2004; Ma and Malynn 2012; Wertz et al. 2015). Studies have demonstrated that A20 acts as a negative regulator that balances the strength and duration of NF-κB activation by deubiquitinating RIP1 (receptor interacting protein 1) and TRAF2 (TNF receptor associated factor 2), the components of TNFR1 signaling complex. Furthermore, the DUB activity of A20 restricts TRAF6-mediated and RIP2-mediated activation of NF-κB during TLR/IL-1R and NOD signaling, respectively (Fig. 3). A20 is also a key inhibitor of T- and B-cell-induced NF-κB signaling. To further regulate cell activation and survival signals, A20 may interact with other proteins that bind to ubiquitin, such as ABIN proteins (A20-binding inhibitor of NF-κB activation), TAX1BP1 (TAX1-binding protein 1), RNF11 (RING-finger protein 11), and IKKγ (NEMO). It remains to be determined how A20 collaborate with these proteins, but it is likely that it functions in larger protein complexes modifying ubiquitin-dependent signaling pathways with a high degree of specificity (Shembade et al. 2010; Ma and Malynn 2012).

The gene encoding A20 (TNFAIP3) is currently qualified as a susceptibility gene for inflammatory disease. Recent human genetic studies strongly associate polymorphisms and mutations in TNFAIP3 with multiple autoimmune and inflammatory diseases, such as rheumatoid arthritis, systemic lupus erythematosus, psoriasis, Crohn’s disease, systemic sclerosis, coeliac disease, type 1 diabetes, inflammatory bowel disease, and coronary artery disease. A20 knockout mice die prematurely due to severe multiorgan inflammation, whereas mice that lack A20 expression in specific immune cell types develop experimental inflammatory diseases, which closely mimic human conditions (Ma and Malynn 2012). As an example may serve A20 ablation in intestinal epithelial cells (IECs) that sensitize mice to dextran sulphate sodium (DSS)-induced colitis and TNF-induced inflammation (Vereecke et al. 2010). Studies revealed that A20 expression in IECs preserves intestinal barrier integrity and mucosal immune homeostasis, which may protect against inflammatory bowel disease in humans (Kolodziej et al. 2011). Independent from its role as a modulator of NF-κB pathway, A20 exerts antiapoptotic activity in several cell types. Being a part of death-inducing signaling complex (DISC), A20 inhibits apoptotic signaling through deubiquitination and inhibition of caspase-8 (Catrysse et al. 2014).

Although some of the studies have shown that the majority of zinc fingers does not respond to changes in free zinc and that deubiquitinase activity of A20 is unaffected by zinc chelator (Haase and Rink 2009), the unusually complex and effective regulation of ubiquitin-depending signals by A20 has been proven to be interdependent with zinc. The induction of A20 mRNA and generation of A20 protein was demonstrated to be zinc-dependent in premonocytic, endothelial, and cancer cells (Prasad et al. 2011). In a study using the HL-60 cells (human promyelocytic leukaemia cell line), zinc enhanced the up-regulation of mRNA and DNA-specific binding for A20, and decreased IL-1β and TNF-α gene expression (Prasad et al. 2004). The results obtained by Prasad suggest that zinc supplementation may lead to down-regulation of the inflammatory cytokines through up-regulation of the negative feedback loop A20 to inhibit induced NF-κB activation. The recent findings confirmed that zinc supplementation influences NF-κB via the alteration of A20 activity. A study by Morgan and colleagues (2011) confirms that zinc is acting on the NF-κB pathway at the level of A20 to further enhance its inhibitory effect. Yan and colleagues (2016) demonstrated for the first time that zinc supplementation prevents abdominal aortic aneurysm (AAA) formation in rats by induction of A20-mediated inhibition of the NF-κB canonical signaling pathway. Li and colleagues (2015) found that zinc contributes to stimulating A20 transcriptional activity via epigenetic modifications at A20 promoter. Moreover, studies demonstrated that physiological state of the cell affects the stability of A20. The protein can be inactivated by reversible oxidation of a key cysteine residue in the catalytic domain in the presence of ROS (Catrysse et al. 2014). Zinc as a free radical scavenger, therefore, also contributes to the enzymatic stability of A20.

Not only A20, but also some other zinc finger-containing proteins may inhibit NF-κB activation. The element is a component of zinc-finger domains of TIZ (TRAF6-inhibitory zinc finger protein), which suppresses TRAF6-induced activation of NF-κB and inhibits the signaling of RANK (receptor activator of NF-κB) (Shin et al. 2002). Correspondingly, zinc increases the expression of peroxisome proliferator-activated receptor α (PPAR-α), which plays an important role in lipoprotein metabolism, inflammation, and glucose homeostasis. PPAR-α inhibits NF-κB activation via negative cross-talk in the nuclear DNA binding level (Reiterer et al. 2004; Bao et al. 2010). The down-regulation of NF-κB activation by zinc via A20 and PPAR signaling pathways is most likely the mechanism by which zinc decreases inflammatory cytokines/molecules including endothelial cell adhesion molecules and oxidative stress biomarkers in atherosclerosis (Bao et al. 2010).

The evidence presented thus far indicates that zinc modulates NF-κB signaling at various levels.

## Concluding remarks

The review provides a brief overview of various mechanisms by which zinc exerts its antioxidant and anti-inflammatory activity. The element does not affect a single component of human immune system. Rather, it influences multiple aspects of the immune system, including haematopoiesis, innate immunity, adaptive immune response, and processes involved in immune regulation. Since impaired zinc homeostasis, constantly increased proinflammatory cytokines and oxidative stress feature prominently in multiple chronic diseases zinc supplementation adjusted to the actual requirement may prove to be a useful preventive and therapeutic agent for human health.
